# The 1-2-3 Rule: When to “Consider” Glaucoma Surgery and Discuss It With Patients

**DOI:** 10.7759/cureus.118768

**Published:** 2026-10-29

**Authors:** Masaki Tanito, Kazunobu Sugihara

**Affiliations:** 1 Department of Ophthalmology, Shimane University Faculty of Medicine, Izumo, JPN

**Keywords:** glaucoma, glaucoma surgery, interventional glaucoma, intraocular pressure, referral timing, shared decision-making, shimane university 1–2–3 rule

## Abstract

Earlier intervention is gaining momentum in glaucoma care, yet delayed referral and delayed consideration of surgery remain concerns in clinical practice. A simple clinical rule may help general ophthalmologists recognize when to discuss procedural options with patients. We propose the Shimane University 1-2-3 rule (SU1-2-3), comprising three prompts: intraocular pressure (IOP) >12 mmHg in progressive advanced glaucoma, with advanced glaucoma defined for this rule as visual field mean deviation ≤ −10 dB or glaucomatous visual field defects within the central 10°; IOP >20 mmHg despite topical treatment in perimetric glaucoma; or use of ≥3 bottles of IOP-lowering eye drops. Oral acetazolamide use is an additional, independent prompt. Meeting any criterion should initiate consideration of surgical options, shared decision-making, and specialist consultation when appropriate, rather than constitute an automatic indication for surgery. SU1-2-3 draws on evidence supporting IOP lowering, treatment-burden assessment, and procedural intervention, but its thresholds and clinical utility have not been prospectively validated. Because its components rely on readily available clinical information, SU1-2-3 may also facilitate consultation when glaucoma subtype classification remains incomplete. It is intended as a minimum safeguard against delayed consideration of surgery, while allowing earlier laser or surgical intervention according to individual needs. SU1-2-3 may serve as a practical clinical and educational reminder for ophthalmologists without glaucoma subspecialty training, general ophthalmologists, and ophthalmology residents.

## Editorial

Momentum is growing toward earlier intervention in glaucoma. The concept of interventional glaucoma, together with an expanding range of laser and surgical options, encourages clinicians to reconsider prolonged escalation of topical therapy [1]. However, this evolving treatment philosophy coexists with a practical challenge: ensuring that patients who may benefit from surgery receive a timely opportunity to discuss it.

In our clinical practice, we encounter patients in whom delayed referral from ophthalmologists who do not perform glaucoma surgery, or delayed surgical intervention after referral, appears to have contributed to poor visual outcomes. Although this observation reflects clinical experience rather than a quantified analysis of referral delays, it highlights the importance of recognizing when a surgical opinion may be valuable. Given the large number of patients with glaucoma and the central role of general ophthalmologists in their care, simple and memorable prompts for this discussion may be useful.

We therefore propose the “Shimane University 1-2-3 rule” (SU1-2-3): intraocular pressure (IOP) >12 mmHg in progressive advanced glaucoma; IOP >20 mmHg despite topical treatment in perimetric glaucoma; or use of ≥3 bottles of IOP-lowering eye drops (Table 1). The institutional designation identifies the origin of this practice-based proposal and is not intended to imply broader endorsement or validation. SU1-2-3 is a heuristic developed at a single institution that awaits prospective validation and should not be cited as a standard of care. For this rule, advanced glaucoma is defined as visual field mean deviation (MD) ≤ −10 dB or glaucomatous visual field defects within the central 10°, and progressive disease refers to documented visual field progression. Perimetric glaucoma is defined as glaucoma with glaucomatous visual field defects. Oral acetazolamide use provides an additional, independent prompt. Any one criterion is sufficient to prompt consideration of surgical options and, when appropriate, referral for a specialist opinion.

**Table 1:** SU1-2-3: when to consider procedural options and discuss them with the patient Progressive disease refers to documented visual field progression. Perimetric glaucoma refers to glaucoma with glaucomatous visual field defects. Any one component prompts consideration and discussion, not an automatic indication for surgery. SU1-2-3 is intended for situations where an individualized target IOP has not yet been established or is unmet, and it does not replace individualized care. Bottle count refers to distinct preparations used to treat the eye under consideration; a fixed-combination preparation counts as one bottle. Rapid progression or suspected acute angle closure warrants prompt assessment irrespective of these thresholds. SU1-2-3, Shimane University 1-2-3 rule; IOP, intraocular pressure; MD, mean deviation

Component	Clinical prompt	Main question for discussion
1	IOP >12 mmHg in progressive advanced glaucoma [2,3]. Advanced glaucoma is defined for this rule as MD ≤ −10 dB or glaucomatous visual field defects within the central 10°.	Should further IOP lowering, including surgical options, be considered in view of ongoing progression in advanced glaucoma?
2	IOP >20 mmHg despite topical IOP-lowering treatment in perimetric glaucoma [4,5].	Can the current treatment strategy achieve an IOP appropriate for an eye with established visual field damage?
3	Use of ≥3 bottles of topical IOP-lowering medication [6].	Is the treatment burden sustainable, and could a procedure help achieve the treatment goals?
Additional independent prompt	Use of oral acetazolamide for glaucoma	What is the intended duration of systemic treatment and the longer-term management plan?

These checkpoints identify when to begin or revisit a discussion with the patient, not when to operate. The discussion should address the current treatment goals, potential benefits and risks of surgery, alternatives, and patient preferences. Its outcome may be continued medical treatment, laser treatment, further assessment, or surgery (Figure 1). SU1-2-3 is intended as a minimum safeguard against delayed consideration of surgery. More proactive intervention may be appropriate before any criterion is met. Documented optical coherence tomography (OCT)-based structural progression may independently warrant treatment escalation or specialist assessment, particularly in preperimetric and early glaucoma, even when visual field progression is not detected, or IOP is below the numerical prompts. The first component retains its focus on functional progression in advanced disease, where some OCT measurements may approach their measurement floor.

**Figure 1:**
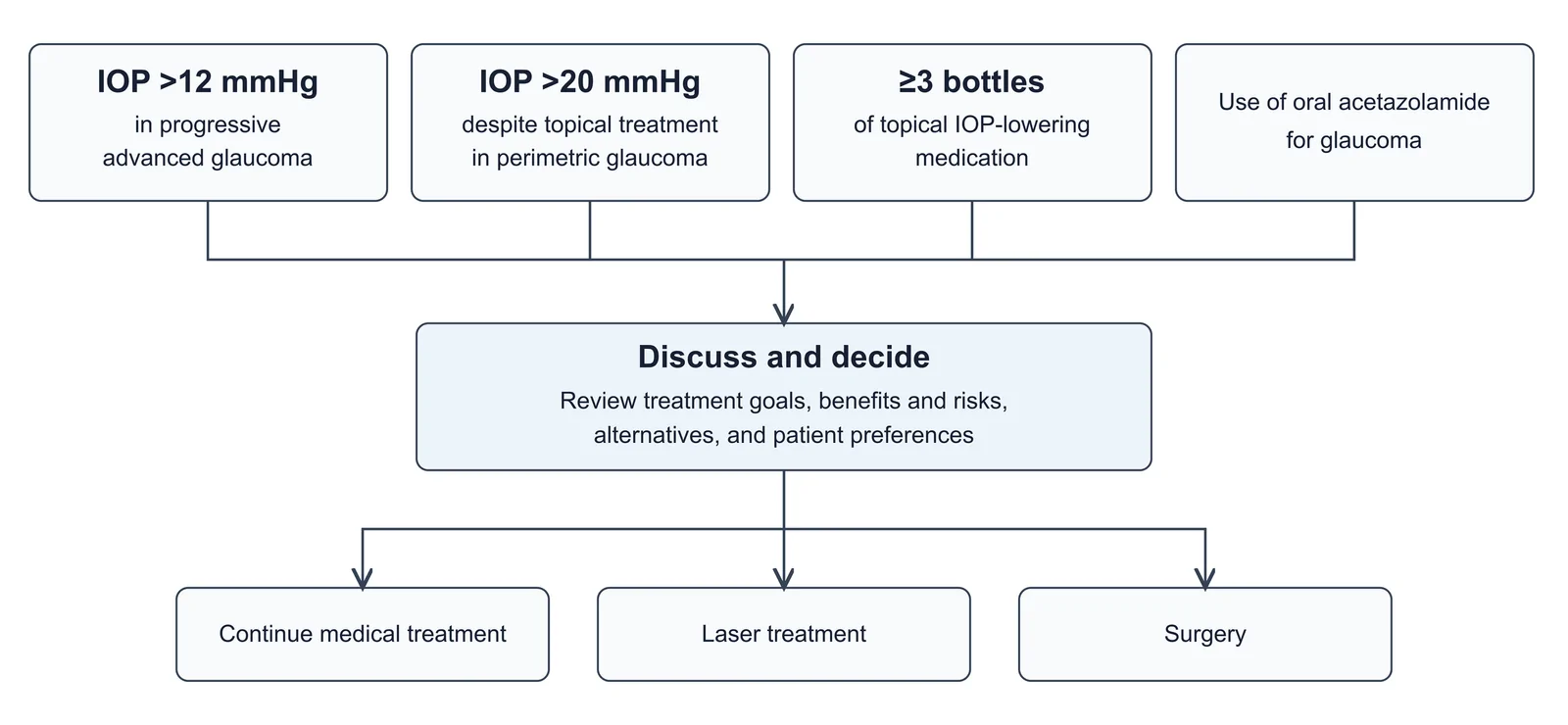
The SU1-2-3 rule: prompts for discussing procedural options in glaucoma Each of the three components—IOP >12 mmHg in progressive advanced glaucoma, IOP >20 mmHg despite topical treatment in perimetric glaucoma, and use of ≥3 bottles of topical IOP-lowering medication—and oral acetazolamide use independently prompts discussion and shared decision-making. The numerical IOP values are heuristic prompts, not evidence-based cutoffs. Discussion incorporates treatment goals, potential benefits and risks, alternatives, and patient preferences, and may lead to continued medical treatment, laser treatment, or surgery. Further assessment remains an option within shared decision-making even though it is not shown as a separate endpoint. Meeting a criterion does not establish an indication for surgery. SU1-2-3, Shimane University 1-2-3 rule; IOP, intraocular pressure

The first component, IOP >12 mmHg in progressive advanced glaucoma, combines advanced disease with evidence that visual function continues to deteriorate. For SU1-2-3, advanced glaucoma is defined as MD ≤ −10 dB or glaucomatous visual field defects within the central 10°. An important purpose of this criterion is to prevent an IOP in the mid-teens from being regarded as satisfactory control solely based on the pressure value, despite ongoing visual field progression in advanced disease. Such apparently reassuring measurements should prompt discussion of further IOP lowering and surgical options when considered alongside the clinical course. In the associative analysis of the Advanced Glaucoma Intervention Study (AGIS), eyes with IOP below 18 mmHg at 100% of scheduled visits over 6 years had a mean IOP of 12.3 mmHg and showed little change in the group mean visual field defect score; this did not mean that every eye remained stable [2]. The Treatment of Advanced Glaucoma Study (TAGS) subsequently randomized 453 patients with newly diagnosed advanced open-angle glaucoma to primary trabeculectomy or initial medical management. At 5 years, the mean IOP was 12.07 and 14.76 mmHg, respectively, and visual field outcomes favored trabeculectomy. Vision-related quality of life did not differ significantly, overall safety-event rates were similar, and serious adverse events were uncommon [3]. The definition of advanced glaucoma used in SU1-2-3 (MD ≤ −10 dB or glaucomatous visual field defects within the central 10°) is a rule-specific operational definition, not a standardized staging classification, and should not be considered equivalent to the definitions or eligibility criteria used in AGIS or TAGS. These findings support considering further IOP lowering in advanced glaucoma but do not establish an IOP cutoff for referral or surgery. We selected 12 mmHg as a pragmatic heuristic informed by the AGIS subgroup mean of 12.3 mmHg and the five-year mean of 12.07 mmHg in the TAGS trabeculectomy arm [2,3]. These are group-level observations, not validated decision thresholds: 12 mmHg is not an evidence-based cutoff, and neither study tested the proposed criterion of IOP >12 mmHg in progressive advanced glaucoma. An IOP at or below this value does not exclude the need for reassessment when progression continues. Progression rate, the location and extent of visual field damage, and anticipated lifetime visual needs remain essential to individual assessment.

The second component, IOP >20 mmHg despite topical treatment in perimetric glaucoma, prompts reassessment of pressure control in an eye with established glaucomatous visual field damage. Unlike the first component, it does not require additional documented visual field progression. Measurement reliability, actual medication use, instillation technique, and reversible causes of IOP elevation should be assessed. The Early Manifest Glaucoma Trial (EMGT) demonstrated the benefit of IOP lowering in 255 patients with early glaucoma and a median baseline IOP of 20 mmHg: laser trabeculoplasty plus topical betaxolol reduced IOP by an average of 25%, and progression occurred in 45% of treated patients versus 62% of initially untreated controls over a median of 6 years [4]. The United Kingdom Glaucoma Treatment Study (UKGTS), a multicenter, triple-masked, placebo-controlled trial involving 516 patients with newly diagnosed open-angle glaucoma, provided complementary evidence. Mean baseline IOP was 19.6 mmHg in the latanoprost group and 20.1 mmHg in the placebo group; at 24 months, the respective mean reductions were 3.8 and 0.9 mmHg. Latanoprost significantly reduced the hazard of visual field deterioration, with an adjusted hazard ratio of 0.44 [5]. Together, these trials show that an IOP around 20 mmHg should not be assumed to provide adequate protection even in early disease, and that further lowering can preserve visual function. Neither trial, however, validates 20 mmHg as a universal target or surgical threshold. In SU1-2-3, 20 mmHg is a heuristic drawn from the baseline IOP levels in EMGT and UKGTS and serves solely as a prompt for reassessment in perimetric glaucoma, not as a treatment target or a threshold for surgical intervention; lower pressures may be required for an individual eye.

The third component, use of ≥3 bottles of IOP-lowering eye drops, addresses treatment burden and the need to reassess continued medication escalation. The fifth edition of the Japan Glaucoma Society guidelines states that treatment alternatives, including glaucoma surgery, should be considered when IOP remains inadequately controlled, or the target IOP cannot be achieved with three topical preparations, including fixed combinations [6]. The guidelines also note inconsistencies in how the underlying studies counted bottles and active agents. SU1-2-3 explicitly uses bottle count, with a fixed-combination preparation counted as one bottle. Bottle count is intended to reflect the practical burden of administering treatment rather than the number of pharmacologically active agents. Although fixed combinations containing multiple agents may not meet this criterion, the number of bottles handled and the frequency of instillation may influence adherence in ways that active-ingredient count alone does not capture. It additionally prompts discussion when measured IOP appears acceptable, allowing the clinician and patient to assess whether the regimen remains sustainable. Dosing frequency, tolerability, instillation ability, and the patient’s experience should inform this assessment. Bottle count alone does not demonstrate poor adherence or establish that surgery is preferable.

Oral acetazolamide use provides a separate reason to review the longer-term treatment plan. A meta-analysis of placebo-controlled studies across several indications found increased risks of paresthesia, taste disturbance, polyuria, and fatigue with acetazolamide [7]. Although these data do not establish a glaucoma-specific surgical threshold, they highlight the importance of considering systemic treatment burden. Short-term use during an acute episode or around surgery differs from continuing maintenance treatment; the discussion should clarify the intended duration and whether a surgical opinion would be helpful.

Accurate subtype diagnosis is important for recognizing potentially blinding forms of glaucoma, including exfoliation glaucoma (EXG) and primary angle-closure glaucoma (PACG). EXG showed faster visual field deterioration than primary open-angle glaucoma (POAG) in a comparative cohort [8], and PACG was associated with a greater burden of blindness than POAG in a meta-analysis, with a pooled risk ratio of 2.39 [9]. However, these subtypes may not be specifically identified in every patient in general ophthalmic practice. Exfoliation syndrome is underdiagnosed, particularly in early disease and after cataract surgery [10], while historical United States studies reported gonioscopy in only 51.3% of patients at the initial visit in 1996 and approximately half of patients in 2007 [11,12]. Although these findings do not establish current practice in Japan, our comparison of referral letters and specialist diagnoses in 515 consecutive patients at Shimane University Hospital also demonstrated substantial differences [13]. Secondary open-angle glaucoma (SOAG) accounted for 20.8% of referral diagnoses and 38.6% of specialist diagnoses, an approximately 1.9-fold difference; EXG constituted 70.4% of specialist-diagnosed SOAG. PACG accounted for 1.9% and 12.0%, respectively, a 6.2-fold difference based on patient counts, while 51.3% of referral letters stated only “glaucoma,” without specifying a subtype. Because SU1-2-3 relies on IOP, visual field findings, and medication burden, it can prompt consultation even when subtype classification remains incomplete.

The broader interventional glaucoma approach supports considering procedural treatment proactively, with attention to medication burden as well as IOP control [1]. In the Laser in Glaucoma and Ocular Hypertension (LiGHT) trial, initial selective laser trabeculoplasty (SLT) was associated with less disease progression and fewer trabeculectomies at six years than initial eye drops [14]. Minimally invasive glaucoma surgery (MIGS) further expands the options for selected eyes. In the HORIZON randomized trial, adding a Schlemm’s canal microstent to cataract surgery increased the proportion of medication-free eyes at five years from 46% to 66% [15]. These findings support discussing alternatives to continued medication escalation, including SLT and, where appropriate, MIGS. However, the results of HORIZON cannot be generalized to every MIGS procedure or to the low IOP requirements of advanced glaucoma, for which TAGS provides evidence supporting primary trabeculectomy [3]. Procedural selection should therefore reflect the required IOP reduction, disease course, ocular anatomy, prior treatment, and patient preferences.

Several limitations should be acknowledged. SU1-2-3 is a practice-based proposal developed at a single institution, and its ability to identify patients who would benefit from surgical assessment has not been prospectively validated. The cited trials and guidelines provide a clinical rationale for its components but do not validate the rule as a whole. None of the cited trials tested a threshold-based referral rule; the proposed components therefore represent pragmatic extrapolations rather than trial-validated referral criteria. The definition of advanced glaucoma used here is an operational definition for SU1-2-3 and is not intended to replace established glaucoma staging systems. Its numerical checkpoints cannot fully capture progression rate, the location and extent of visual field damage, lifetime risk of visual impairment, or patient preferences. The first component also depends on detecting visual field progression, which may be difficult when reliable longitudinal data are limited. Bottle count is an imperfect measure of treatment burden because formulations and dosing schedules vary. Bottle count may under-capture treatment burden when multiple fixed combinations are used. Consequently, patients who do not meet these criteria may still require prompt specialist assessment, whereas meeting a criterion does not establish that surgery is appropriate. Confirmation of IOP by repeated measurements, with assessment of diurnal variation when clinically indicated, is desirable to inform management decisions. However, because this rule is intended primarily for clinicians without glaucoma subspecialty training, completion of these assessments is not a prerequisite for referral. A clinician may seek a specialist opinion on the basis of an initial IOP measurement considered alongside the clinical findings, with further confirmation undertaken during specialist assessment. Such referral does not imply that a single measurement establishes an indication for intervention. The effects of SU1-2-3 on referral patterns, clinical workload, treatment burden, and visual outcomes require evaluation.

SU1-2-3 may be particularly useful for ophthalmologists without glaucoma subspecialty training, general ophthalmologists, and ophthalmology residents as a simple clinical and educational reminder of when to consider surgical options and discuss them with patients. The rule is intended primarily to prompt specialist assessment when an individualized management plan, including a target IOP, has not yet been established. It is not intended to replace or override ongoing individualized care guided by an established target IOP and assessment of disease progression. By using readily available information, it may also support timely consultation when subtype classification remains incomplete. Referral for a surgical opinion offers an opportunity for assessment and shared decision-making without implying a commitment to an operation. The purpose of SU1-2-3 is to help initiate that conversation while useful vision can still be preserved, alongside the continuing movement toward earlier intervention.
